# Scanning Electrochemical Impedance Microscopy in Redox-Competition Mode for the Investigation of Antibodies Labelled with Horseradish Peroxidase

**DOI:** 10.3390/ma14154301

**Published:** 2021-07-31

**Authors:** Antanas Zinovicius, Inga Morkvenaite-Vilkonciene, Almira Ramanaviciene, Juste Rozene, Anton Popov, Arunas Ramanavicius

**Affiliations:** 1 Department of Mechatronics and Robotics, Vilnius Gediminas Technical University, J. Basanavičiaus 28, LT-03224 Vilnius, Lithuania; antanas.zinovicius@vilniustech.lt (A.Z.); juste.rozene@vilniustech.lt (J.R.); 2 Laboratory of Electrochemical Energy Conversion, State Research Institute Centre for Physical Sciences and Technology, Sauletekio 3, LT-02300 Vilnius, Lithuania; 3 NanoTechnas—Center of Nanotechnology and Materials Science, Faculty of Chemistry and Geosciences, Vilnius University, Naugarduko 24, LT-03225 Vilnius, Lithuania; almira.ramanaviciene@chf.vu.lt (A.R.); anton.popov@chgf.vu.lt (A.P.); 4 Department of Physical Chemistry, Institute of Chemistry, Faculty of Chemistry and Geosciences, Vilnius University, Naugarduko 24, LT-03225 Vilnius, Lithuania; arunas.ramanavicius@chf.vu.lt; 5 Laboratory of Nanotechnology, Department of Functional Materials and Electronics, State Research Institute Centre for Physical Sciences and Technology, Sauletekio 3, LT-02300 Vilnius, Lithuania

**Keywords:** scanning electrochemical microscopy, antigen-antibody complex, horseradish peroxidase, immunoassay, electrochemical methods, electrochemical impedance spectroscopy, bioelectrochemistry, bioelectronics, ultra-micro electrode, scanning electrochemical impedance microscopy

## Abstract

Scanning electrochemical microscopy enhanced by electrochemical impedance spectroscopy (SEIM) was applied to detect immobilized antibodies labelled with horseradish peroxidase (Ab-HRP). The localized HRP activity was investigated by the SEIM redox competition (RC-SEIM) mode using hydrogen peroxide as a substrate and hexacyanoferrate as a redox mediator. Electrochemical impedance shows to be related to the consumption of hydrogen peroxide at the ultramicroelectrode. For the evaluation of impedimetric results, an equivalent electric circuit was applied with solution resistance, double-layer capacitance, and charge-transfer resistance. These equivalent circuit characteristics depend on the distance between the sample and ultramicroelectrode, and the concentration of substrate. From the gathered data, the charge-transfer resistance appeared to be the parameter describing the behavior of HRP catalyzed reaction as it showed a linear dependence on H_2_O_2_ concentration. The RC-SEIM mode suitability for the studying of HRP catalyzed reactions and for the evaluation of Ab-HRP bound to the surface was demonstrated. Additionally, the applicability of RC-SEIM mode for the determination of Ab-HRP affinity bound to the target analyte was discussed.

## 1. Introduction

Scanning electrochemical microscopy (SECM) is widely applied to evaluate enzymatic assays. However, for the evaluation of antibody and antigen interaction it is rarely used [1]. SECM allows performing the evaluation of electrochemical processes that occur on surfaces. In addition, it is suitable to gain some quantitative data about electrochemical characteristics of surfaces and the presence of active antigen binding sites in the layer of immobilized antibodies [2,3]. Moreover, SECM allows the development of miniaturized analytical devices. However, both qualitative and quantitative analyses of some biologically active analytes (antibiotics, hormones, cytokines, etc.) are still rather complicated. Moreover, the detection quality of formed antibody and antigen immune complex decreases with the molecular weight of the antigen [4]. Furthermore, a significant influence of foreign substances on the analytical signal has been noticed from working with the real samples. Additionally, better results were obtained using labelled analytes [3,5] or antibodies [6] during the analyses. Alkaline phosphatase and horseradish peroxidase (HRP) are often used for antibody (or antigen) labelling, in which the activity was successfully investigated by SECM [7,8,9]. HRP is one of the best candidates for labelling of antibodies due to the small size, fast enzymatic reaction (for instance, compared with glucose oxidase), and the possibility to use a wide range of redox mediators (for instance, compared with alkaline phosphatase). HRP-labelled antibodies are frequently used in Western blotting, immunohistochemistry, and enzyme-linked immunosorbent assay (ELISA) [10,11,12]. 

SECM and electrochemical impedance spectroscopy (EIS) techniques can be applied together as scanning electrochemical impedance microscopy (SEIM), which could allow performing a sensitive electrochemical detection of low molecular weight analytes without applying potential [13,14]. In SEIM, electrochemical impedance spectra are recorded on all points of interest of 3D space and the sample can be evaluated by applying the corresponding equivalent electrical circuit. A typical EIS can also be applied for the evaluation of antibody and antigen interaction occurring on the electrode surface [15,16,17,18,19]. However, the sample must be prepared by detecting the electrode, and this issue generates additional problems, such as: The immobilization of biomolecules and washing steps occur on the same surface, e.g., detecting the electrode; the biomolecules slow down the charge transfers and the diffusion of redox species; detecting the electrode can be used more than once only after the efficient regeneration procedure; and the biological activity can be decreased due to the biomaterial contact with the conductive support. On the contrary, SECM allows locally detecting the biomolecules immobilized on both conductive and non-conductive supports. In this case, the processes occurring on the support can be detected by the ultramicroelectrode (UME) that does not touch the surface of interest. Therefore, the biomolecules are not affected, the diffusion of redox species is not blocked, and UME can be used many times. As a result, SECM seems to be a more powerful tool for the development of biosensing systems. 

Earlier in our research, we applied SEIM to evaluate the conductivity of the solution at a close distance to the enzyme, where an enzyme-catalyzed reaction occurs [20]. Furthermore, to increase the sensitivity evaluating enzyme-modified surfaces, SECM in the redox competition (RC-SECM) mode was used [21]. The RC-SECM mode relays on the competition between the UME and enzyme-modified surface for the same redox species. It was found that using the RC-SECM mode, the smaller concentrations of the analyte can be detected and results can be evaluated by mathematical models [22,23,24].

In this research, the RC-SECM mode and EIS were used to achieve better sensitivity for the detection of a low amount of HRP-labelled antibodies (Ab-HRP) bounded to the surface. Additionally, the applicability of the SEIM in redox competition (RC-SEIM) mode for the determination of Ab-HRP in real-time was demonstrated. To the best of our knowledge, the RC-SEIM mode for the assessment of immobilized Ab-HRP was used for the first time.

## 2. Materials and Methods

### 2.1. Materials

Ab-HRP was obtained from the ELISA kit produced by Institut Pourquier (Montpellier, France). Hydrogen peroxide, K_4_[Fe(CN)_6_] K_3_[Fe(CN)_6_] was delivered by Carl Roth GmbH (Berlin, Germany). In addition, 25% of glutaraldehyde solution was delivered by Fluka Chemie GmbH (Buchs, Switzerland). Moreover, 98.5% of ethanol solution from “Vilniaus degtinė” (Vilnius, Lithuania) was used for electrodes and plastic cells cleaning. 

The supporting electrolyte for the electrochemical experiments was 0.1 M acetate- phosphate buffer (A-PBS), pH 6.5, which was prepared using NaH_2_PO_4_ from Fluka Chemie GmbH (Bucharest, Romain), Na_2_HPO_4_ from Carl Roth GmbH (Berlin, Germany), KCl from Scharlau (Barcelona, Spain), and CH_3_COONa from Merk (Tokyo, Japan). The pH was adjusted with CH_3_COOH from Merk (Darmstadt, Germany) or NaOH from Merk (Steinheimm, Germany).

### 2.2. Immobilization of Ab-HRP

A petri dish made of poly(methyl methacrylate) was cleaned by 98.5% of ethanol, dried, and kept over a 25% solution of glutaraldehyde for 15 min in order to activate the surface with glutaraldehyde, which is necessary for covalent Ab-HRP immobilization. Afterwards, 1 µL of 40 mg/mL Ab-HRP was loaded on the surface and dried at room temperature. After this, 25% of glutaraldehyde solution was again used in the same way to cross-link Ab-HRP attached to the surface and rinsed with the A-PBS solution.

### 2.3. Evaluation of Ab-HRP-Modified Surface by Electrochemical Impedance Spectroscopy 

**Figure 1:**
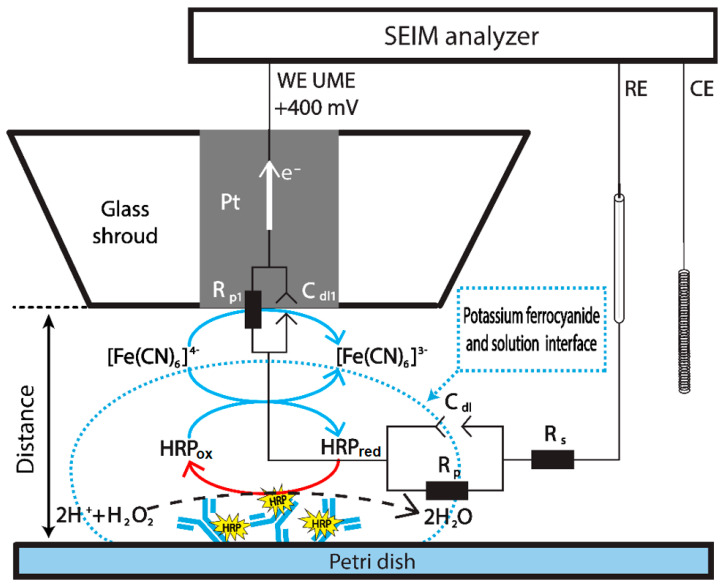
Schematic representation of Ab-HRP detection by EIS in the presence of potassium ferrocyanide (K_4_[Fe(CN)_6_]), which serves as a redox mediator. HRP_ox/red_—oxidized and reduced forms of horseradish peroxidase.

EIS measurements were performed using three electrodes of an electrochemical cell. The platinum UME with 10 µm radius from Sensolytics GmbH (Bochum, Germany) was connected as a working electrode, a platinum wire as a counter electrode, and Ag/AgCl as a reference electrode. The distance between the UME and sample surface was determined from the registered current vs. distance dependencies while approaching UME to the plastic surface in 1 mM K_3_[Fe(CN)_6_]/K_4_[Fe(CN)_6_] solution. EIS was measured at 20 kHz to 100 mHz; 10 mV of RMS amplitude; and DC potential of +400 mV. Measurements were performed in every step approaching the Ab-HRP-modified surface. The scan speed of UME was 1 µm/s. The equivalent circuit of potassium ferrocyanide and solution interface is shown in Figure 1. The measured electrochemical impedance is expressed by the following equation:

(1) $$Z=\frac{Z_{Cdl}\cdot \left(R_{p}\right)}{Z_{Cdl}+\left(R_{p}\right)}+R_{s}$$

where $Z_{Cdl}$ is the impedance of double-layer (Equation (2)), $R_{p}$ is the resistance of charge-transfer, and $R_{s}$ is the ohmic resistance of the solution.

Double-layer impedance is expressed as:

(2) $$Z_{Cdl}=\frac{1}{Q\;{\left(j\omega \right)}^{\alpha }}$$

where *Q* is the capacitance, *j* is the imaginary unit, *ω* is the angular frequency, and *α* is the empirical constant.

## 3. Results and Discussion

The oxidation of [Fe(CN)_6_]^4−^ is possible on the surface of UME or during the enzymatic HRP reaction on the support with immobilized Ab-HRP (Figure 1). Therefore, the competition between the enzyme and UME electrode for this form of redox mediator occurs. In this way, the UME registers the consumption of [Fe(CN)_6_]^4−^, in which the concentration is related to the activity of HRP present in Ab-HRP conjugate. When the distance between the UME and Ab-HRP-modified surface increases, the current, which is directly proportional to the [Fe(CN)_6_]^4−^ concentration, decreases. The registration of EIS locally allows us to explore the diffusion profile of [Fe(CN)_6_]^4−^.

The reaction on the UME [25]:

(3) $$2{\left[\mathrm{Fe}(\mathrm{CN}{)}_{6}\right]}^{4-}\;-\;2e^{-}\;\overset{+0.4\;V}{\to }\to \;2{\left[\mathrm{Fe}(\mathrm{CN}{)}_{6}\right]}^{3-}$$

The reaction, catalyzed by HRP:

H_2_O_2_ + 2[Fe(CN)_6_]^4−^ + 2H^+^ → 2H_2_O + 2[Fe(CN)_6_]^3−^ (4)

**Figure 2:**
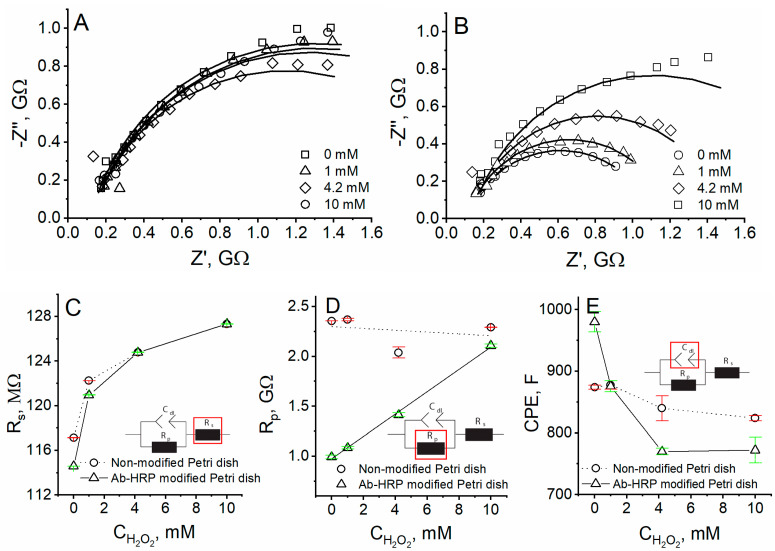
Nyquist plots of experiments performed at 2-µm distance from (**A**) unmodified Petri dish and (**B**) Ab-HRP- modified Petri dish in A-PBS with 1 mM K_3_[Fe(CN)_6_]/K_4_[Fe(CN)_6_] using different concentrations of H_2_O_2_; dependence of (**C**) resistance of solution, (**D**) resistance of charge-transfer, (**E**) double-layer capacitance on H_2_O_2_ concentration using unmodified and Ab-HRP-modified Petri dishes.

**Figure 3:**
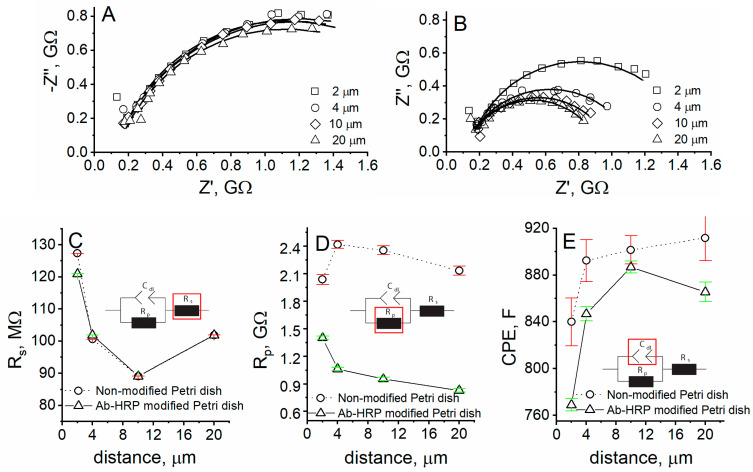
Nyquist plots of experiments performed at different distances from (**A**) unmodified and (**B**) Ab-HRP-modified Petri dishes in APBS with 1 mM solution of K_3_[Fe(CN)_6_]/K_4_[Fe(CN)_6_] using 4.2 mM H_2_O_2_ concentration; dependence of (**C**) resistance of solution, (**D**) resistance of charge-transfer, (**E**) double-layer capacitance on distance between the UME and surface of Petri dish.

The EIS spectra were registered at different H_2_O_2_ concentrations (Figure 2) and different distances (Figure 3) from the unmodified or Ab-HRP-modified Petri dish surface. Nyquist plots, observed at 2-µm distance from the unmodified Petri dish, show minimal changes at different H_2_O_2_ concentrations (Figure 2A). However, Nyquist plots of impedance at 2-µm distance from the Ab-HRP immobilized on the surface of Petri dish, show that the charge-transfer resistance increases with the H_2_O_2_ concentration (Figure 2B). Therefore, the Ab-HRP can be detected by SEIM in RC-SEIM mode. Moreover, the activity of HRP can be investigated by increasing the concentration of the substrate.

The Randles circuit (Equations (1) and (2)) was fitted to the EIS data, and all of the three parameters were plotted as the H_2_O_2_ concentration function. The measured solution resistance was the same for both unmodified and Ab-HRP-modified Petri dishes using various H_2_O_2_ concentrations (Figure 2C). In the case of unmodified Petri dish, the charge-transfer resistance dependence on the H_2_O_2_ concentration was not registered, wherein the linear dependence was determined when Ab-HRP was immobilized on the surface (Figure 2D). The double-layer capacitance decreases when the H_2_O_2_ concentration increases up to 4.2 mM and remains unchanged at 10 mM (Figure 2E). The charge-transfer resistance linearly depends on the H_2_O_2_ concentration, thus, the Ab-HRP-modified surface can be investigated in RC-SEIM mode.

To investigate the method of RC-SEIM for the research of Ab-HRP-modified surface, EIS spectra were registered at different distances from unmodified and Ab-HRP-modified Petri dishes. The EIS spectra, registered at different distances from the unmodified Petri dishes, as it was expected, showed no changes (Figure 3A). The charge-transfer resistance of the EIS spectra registered at the Ab-HRP-modified Petri dish is higher at lower distances due to the consumption of [Fe(CN)_6_]^4−^ and hindered diffusion to UME (Figure 3B). The solution resistance was found to be the same for unmodified and Ab-HRP- modified Petri dishes at all measured distances (Figure 3C), while the charge-transfer resistance dependence on distance is of an entirely different behavior at unmodified and Ab-HRP-modified Petri dishes (Figure 3D). The charge-transfer resistance was from 1.5 times (at 2-µm distance) to 2.6 times (at 20-µm distance) lower for the Ab-HRP-modified surface, compared to the unmodified Petri dish. Moreover, the behavior of charge-transfer resistance dependence on distance was similar to the SECM approach curves, registered in negative feedback or RC-SECM mode. It means that when approaching the surface, the [Fe(CN)_6_]^4−^ concentration becomes lower, thus it is a clear evidence of the consumption of redox mediator at the surface pre-modified with Ab-HRP.

## 4. Conclusions

The RC-SEIM mode was applied to detect enzyme HRP-labelled antibodies immobilized on the surface of Petri dishes. EIS spectra were registered at different H_2_O_2_ concentrations and different distances from the empty Petri dish or modified with Ab-HRP. It was observed that the Ab-HRP can be detected by SEIM in RC-SEIM mode, and the activity of HRP can be investigated by increasing the concentration of the substrate. The Randles circuit was fitted to the EIS data, and all of the three parameters were plotted as a function of H_2_O_2_ concentration or distance. The charge-transfer resistance R_p_ seems to be the parameter, which can describe the behavior of HRP-catalyzed reaction at the surface, substrate consumption, and the diffusion profile. The charge-transfer resistance linearly depends on the H_2_O_2_ concentration, thus, the Ab-HRP-modified surfaces can be investigated by the RC-SEIM mode. The charge-transfer resistance is from 1.5 (at 2-µm distance) to 2.6 times (at 20-µm distance) lower for Ab-HRP, compared to the Petri dish. Moreover, the behavior of charge-transfer resistance dependence on distance is similar to the SECM approach curves, registered in negative feedback or RC-SECM mode.

These findings show that the RC-SEIM mode can be successfully applied in immunoassays for different analytes detection after affinity interaction with HRP-labelled antibodies.
